# A fragile but critical link: a commentary on the importance of government-academy relationships

**DOI:** 10.1186/s13584-018-0247-7

**Published:** 2018-10-02

**Authors:** Adalsteinn Brown, Greg Marchildon, Stephen Bornstein, Moriah Ellen

**Affiliations:** 1 Dalla Lana School of Public Health, University of Toronto, Sixth Floor, 155 College Street, Toronto, ON M5T 3M7 Canada; 2 Institute of Health Policy, Management and Evaluation, Dalla Lana School of Public Health, Toronto, Canada; 3 Department of Obstetrics and Gynecology, Faculty of Medicine, University of Toronto, Toronto, Canada; 4 Centre of Applied Health Research, Memorial University of Newfoundland, St. John’s, NF Canada; 5 Department of Political Science, Memorial University of Newfoundland, St. John’s, NF Canada; 6 Faculty of Medicine, Memorial University of Newfoundland, St. John’s, NF Canada; 7 Department of Health Systems Management, Guilford Glazer Faculty of Business and Management and Faculty of Health Sciences, Ben-Gurion University, Beersheba, Israel

## Abstract

Interactions between government and academia can be an important support to effective policy and they can also ground researchers’ methods and perspectives more strongly in the realities of policy-making and politics, leading to more relevant research. If properly developed, these interactions can lead to relationships between government and academia that re-enforce evidence-informed policy and useful research. However, strong relationships require repeated interactions and strong personal connections, something that can be supported through careers that cross academia and government. Academic and public service polices that value these kinds of careers can help build strong relationships.

Like Glied, Wittenberg, and Israeli, three of us have spent time working both as academics and as senior government officials, although in Canada rather than in Israel, the UK or the US. This brings a different social and political context into play. And, in contrast to the largely one-way flow from academia to government [1] that the authors describe, we have moved in both directions. However, our career paths are unusual: in Canada, the majority of government-university interactions follow the paths described by Glied and colleagues where there is either a carefully constructed interaction, limited in time or nature, or movement from academia to government but not back again. Indeed, we know of more than a few colleagues who left the academy permanently after taking on a senior role in government.

As in other jurisdictions like the US, Canadian researchers tend to have carefully defined sets of interactions with government that involve maintaining a permanent home within the University while taking on secondments, working on advisory committees or participating in directed research projects where government articulates the question (the “what”) but leaves the academics free to determine the methods (the “how”) and to publish the results in typical scholarly format. This third form of interaction, in which researchers focus some portion of their scholarly effort on questions selected by health policy-makers, has a particularly long and vigorous history in Canada. Building on the integrated knowledge transfer model developed by Graham and colleagues [2], Canadian funding agencies have developed programs like the now-defunct Partnerships for Health System Improvement that required decision-makers to provide partnerships and endorsements, or the current Innovative Clinical Trials Competition. These programs bring together researchers and decision-makers with the promise of a financial payback model from the returns to successfully implemented health services research.

As Glied and colleagues convincingly argue, government-university interactions are vital to effective policy and they can also ground researchers’ methods and perspectives more strongly in the always complex and sometimes gritty realities of policy-making and politics. Thus, these interactions are valuable to both government and the academy. In this commentary, we will focus in on two characteristics of these bilateral relationships that can make them more understandable and potentially more valuable.

The first characteristic is the density and strength of the relationships themselves. Several years ago, Jonathan Lomas worked as an embedded scholar in the Ontario Ministry of Health and Long-term care, evaluating and critiquing that Ministry’s efforts to use evidence [3]. Based on this experience, he argued for the importance of both push and pull mechanisms to increase the use of evidence. That is, policy-makers needed to seek out and commission (“pull”) evidence and researchers needed to advocate and package their research so as to facilitate consumption (“push”) by policy-makers. He also advocated more extensive and sustained relationships between researchers and policy-makers as the conduit through which both push and pull could be increased.

More recently, two of us [4] argued from a game-theory perspective that these relationships could be improved by increasing their frequency. Although this might seem like a thinly veiled argument for more or larger grant funding programs – albeit ones that rely on some version of the Integrated Knowledge Transfer Model – it is actually an argument for more of the types of interactions described by Glied and colleagues. Long-lasting relationships, both formal and informal, are a crucial factor in the use of research to inform policy. Numerous frameworks discuss the importance of linkage and exchange between academics and decision makers, yet the actualities and technical aspects are not fully explored in the research literature. We postulate that country context and working climate have a strong influence. Furthermore, in our experience, actual working relationships in which academics spend time in government, or in which bureaucrats spend time working in a university, or in which the two collaborate through committees or projects that force them to work together on an ad-hoc basis, are much more likely to build strong relationships than grants that require a decision-maker’s endorsement as a condition of funding and some type of (often and unfortunately) passive knowledge transfer at its conclusion.

This takes us to the second point that we want to emphasize. Glied et al. conclude that “government service is both intellectually and personally rewarding.” This fact is far too often neglected or undersold in the academy. Yet, in Canada and likely in many other countries, the university and the public service sit professionally as two solitudes largely because the two institutions have very different incentive systems.

Once they are inside the public service, the pressure for diverse experiences within government and the importance of long service for promotion mean that few public servants can leave government for an extended period of time without some risk to their long-term career prospects. Similarly, public servants who spend any extended period of time in one ministry, such as a ministry of health, may feel pressured to move laterally into a non-health ministry in order to move up in rank. This can undermine the relationships they have been building with scholars. It can also encourage a generalist approach in the public service that can be problematic if it becomes the dominant mode of expertise [5]. Likewise, the increasing scarcity of tenure-track positions and the requirement of a high and unrelenting level of publication and grant success for academic advancement mean that few academics can contemplate leaving an academic post, even temporarily. This is particularly the case in the early years of their careers, when a stint in government might actually be most valuable for shaping their perspectives and building long-term relationships.

Thus, the logics of success in both the academy and bureaucracy militate against the establishment of long-term relationships that value expertise within a specific sector like health and that are built by public servants and academics working together. The only way we can see to counter this is to re-enforce Glied, Wittenberg, and Israeli’s argument that each sector needs to change its incentive system to regard time spent in the other as a valuable part of a career. This is particularly true in the area of health systems and policy, where research is best applied to relevant policy problems, and evidence-informed decision-making is critical to those who steward and manage our health systems.

So, what are some of the practical ways to enhance the quality of the relationship for both government and the academy? Based on our respective experiences, we identify some promising avenues.

A recent program funded by the Canadian Institutes of Health Research (CIHR) and called the Health System Impact Fellowships, embeds recent PhD graduates in ministries, public agencies, healthcare providers and even the private sector as a way of exposing recent PhD graduates to the value of alternative career pathways. Although only in its second year, it will soon have a cohort of nearly 100 fellows who may provide the impetus necessary to encourage this shift in mindset both in the academy and in the public service. At some point, this program should undergo a rigorous external evaluation so that the merits and demerits of such an approach can be assessed both in Canada and in other countries, especially those without similar programs.

We should also encourage the development of more academic networks with government partners and mixed advisory boards. Networks such as the two-decade-old European Observatory on Health Systems and Policies [6] and the more recent Asia Pacific Observatory on Health Systems and Policies [http://www.searo.who.int/asia_pacific_observatory/en/] and the North American Observatory on Health Systems and Policies [http://ihpme.utoronto.ca/research/research-centres-initiatives/nao] regularly bring independent academics into direct contact with public servants and their ministers to work on policy problems identified by decision-makers. The working approach of these observatories relies heavily on leveraging (and mixing) the different incentive systems that shape behaviours in government and the academy.

Lastly, we should also explore the possibility of leveraging already existing mechanisms to enhance these relationships. For example, in Canada, through CIHR, there have been many different funding opportunities that have required partnerships between researchers and decision makers. However, too often these partnerships involve only initial endorsement and passive interaction. Sibbald and colleagues argue that funding agencies can enhance these partnerships by ensuring that certain factors are in place to support these partnerships such as regular, multifaceted, two-way communication, partnerships building on existing relationships, and alignment of agendas [7].

## Conclusions

While these are just some examples of practical approaches that can support the relationships between academic and policy, we recognize that these approaches may not be the best approach in every country and in every context. However what we do think is essential in every country, as well as in every context, is building strong, trusting, long-term relationships between those that actively produce research with those that ultimately should be using the research. The ‘how’ may change between countries and contexts, but the ‘what’ is essential across all borders.
